# Daylight and School Performance in European Schoolchildren

**DOI:** 10.3390/ijerph18010258

**Published:** 2020-12-31

**Authors:** Ramen Munir Baloch, Cara Nichole Maesano, Jens Christoffersen, Corinne Mandin, Eva Csobod, Eduardo de Oliveira Fernandes, Isabella Annesi-Maesano, on behalf of the SINPHONIE Consortium

**Affiliations:** 1 Epidemiology of Allergic and Respiratory Diseases (EPAR) Department, Saint-Antoine Medical School, INSERM, Pierre Louis Institute of Epidemiology and Public Health (IPLESP UMRS 1136), Sorbonne Université, 27 Rue de Chaligny, CEDEX 12, 75571 Paris, France; cara.maesano@iplesp.upmc.fr (C.N.M.); isabella.annesi-maesano@inserm.fr (I.A.-M.); 2 Velux A/S, Ådalsvej 99, DK-2970 Hørsholm, Denmark; Jens.Christoffersen@velux.com; 3 Scientific and Technical Centre for Building, University Paris Est, 77447 Marne-la-Vallée, France; corinne.mandin@cstb.fr; 4 Regional Environmental Center for Central and Eastern Europe (REC), 9–11 Ady Endre ut, 2000 Szentendre, Hungary; ecsobod@rec.org; 5 Institute of Science and Innovation in Mechanical Engineering and Industrial Management (INEGI), 4200-465 Porto, Portugal; eof@fe.up.pt

**Keywords:** illuminance, natural light, indoor air quality, schools, performance

## Abstract

Aims and objectives: Lighting constitutes a critical issue in school design because of its importance as a strong enabler of performance, which is crucial for child development. However, data on light impacts on school performance are scarce. The main objective of this study was to assess the relationship between daylighting conditions in classrooms and mathematical and logical test scores. Methods: The population-based SINPHONIE (Schools Indoor Pollution and Heath: Observatory Network in Europe) study provides information on relationships between lighting conditions and school performance for 2670 elementary schoolchildren, aged 8–13 years from 155 classrooms in 53 schools across 12 European countries. These data were acquired through direct physical assessments and questionnaires completed by teachers, schoolchildren, and their parents, allowing for estimations of multiple objective daylight indicators, as well as subjective parameters such as the perception of lighting. Schoolchildren performed an attention/concentration exam that included simple mathematical exercises in addition to a logical ciphering test. The corresponding performance scores were compared against multiple daylighting parameters. Results: A positive relationship was found between performance scores and type of window shading, latitude, percentage of window facing south, and window glazing, with the highest impact due to the window-to-floor area ratio. Conclusion: Data collected in the SINPHONIE study across 12 European countries indicate that daylighting parameters are relevant to schoolchildren’s performance. As SINPHONIE was not designed specifically with lighting in mind, dedicated studies covering a wide range of classroom configurations would be enlightening.

## 1. Introduction

Light constitutes a critical issue in building design because of its importance as a strong enabler of performance. A recent review on lighting conditions in the work environment showed that specific illuminance conditions may promote better performance [1]. This is of the upmost importance in school buildings, where small differences in academic performance can become amplified differences in economic success later in life [2]. Other important factors that can determine educational and economic prosperity are school satisfaction, health behavior and subjective wellbeing. Given that light is an important factor in human physiology, mental health, and behavior, it is worth continuing to investigate its effects on human performance. In particular, daylight has also been advocated as a means to reduce energy consumption in buildings [3].

Few studies have tried to establish a link between light and school performance in children in population-based studies, and results have shown the beneficial effect of daylight on performance [4,5,6,7,8,9,10,11,12]. Some studies have suggested that poor lighting in school classrooms can negatively affect both children’s health and their ability to learn [13]. Other studies have shown that both sufficient natural sunlight and/or dynamic lighting systems can improve learning [14]. Dynamic lighting has been defined as lighting that aims to impact potential effects such as color temperature and illuminance. Recently, a field study among office workers suggested no significant difference between health effects such as headache, eyestrain, alertness, and dynamic and static lighting, but extrapolation to children is not possible [15]. In another recent field study conducted on a small sample, the combination of dynamic lighting and increased ventilation rate indicated a boosted positive impact on the speed and concentration of the children, suggesting that future renovations would benefit from a holistic design including both of these factors [16]. Overall, inconsistencies exist among the results obtained in the existing studies. Such inconsistent findings may partly depend on the study designs, given that parameters such as number and age of participants, type of lighting, and duration vary greatly between studies, some being interventional studies and others being cross-sectional in design [12]. Lastly, daylight may limit the potential harmful effects of artificial light exposure [13,14].

The primary objective of the present study was to investigate whether schoolchildren’s performance is affected by daylighting conditions in the classroom. This was attained by estimating schoolchildren’s exposure to light through the assessment of various classroom characteristics, as well as through personal perception of light, and by relating these characteristics to performance tests in the context of the European SINPHONIE study, which was conducted at the general population level using a standardized protocol. The study protocol allowed for the control of other known predictors of schoolchildren’s learning, including health, social status, and classroom and air quality.

## 2. Material and Methods

### 2.1. Study Design and Population

The SINPHONIE (Schools Indoor Pollution and Heath: Observatory Network in Europe), a population-based study conducted in Europe, was conducted over the period 2011–2012 to investigate the environmental impacts of schools and classrooms on the health and performance of schoolchildren [17]. The project covered 115 schools and 319 classrooms across 23 European countries. While the SINPHONIE study did not prioritize lighting conditions over other environmental concerns, such as indoor pollution estimates, their dataset does include enough information about the daylight conditions for each classroom to warrant a detailed investigation into their effect on school performance.

Out of the 23 participating countries, 12 conducted performance exams, for a total of 2670 schoolchildren, aged 8–13 years. Over half of the schoolchildren were Caucasian (65.54%), and there were approximately equal numbers of male and female schoolchildren. The countries were grouped into four geographical clusters sharing similar characteristics according to the World Health Organization (WHO): Cluster 1 (Northern Europe), Cluster 2 (Western Europe), Cluster 3 (Central-Eastern Europe), and Cluster 4 (Southern Europe) (Table S1).

### 2.2. Light Assessment

Data were acquired through both direct physical measurements as well as by the use of questionnaires. Teachers, schoolchildren, and parents were all given questionnaires to assess the conditions at the school, the health of the schoolchildren, and the perception of air quality. Parameters that could be objectively quantified, such as window and classroom size, were measured and recorded by an outside surveyor.

Variables considered to have a possible influence on the amount of light inside the classroom included in the SINPHONIE study were the following: type of lighting (natural, artificial, or mixed), window size, presence of direct sunlight, type of window glazing, type of shading, latitude, percentage of windows facing south, and openable windows. Additional variables were created to enhance the capabilities of the data provided and were obtained by combining variables or including geographical information. The window-to-floor ratio is the ratio of measured window area to measured floor area and is used to understand how “big” the windows are compared to the size of the room. All variables relating to light are detailed in Table 1, which lists the collected variables and created variables respectively. The wall covering, type of blackboard, type of lighting (artificial, natural, or a combination) or window glazing (single, double, triple, double clear with filling), and shading (internal, external, none) have multiple options, as listed in Table 1. CO_2_ measurements are also included as a confounding variable.

**Figure 1:**
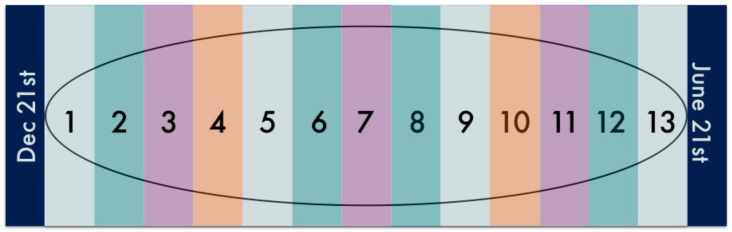
Daylight Indices as a measure of daylight hours. The ellipse represents a full calendar year. The closest 4 weeks (7 December–3 January) surrounding 21 December are the darkest and correspond to Index 1. The next closest 2-week periods (23 November–6 December and 4–17 January) correspond to Index 2, and so on. The brightest 4 weeks of the year correspond to Index 13.

The Daylight Index was created as a relative measure of daylight hours and is intended to replace season or month as a measure of relative daylight (Figure 1). To calculate the daylight index, we split year into 13 equal time periods, each containing four weeks with approximately the same amount of daylight hours. It should be noted that these are not necessarily four consecutive weeks but rather two two-week periods around the solstices. Daylight Index 1 represents the four darkest weeks of the year surrounding the Winter solstice. The 2-week periods of 23 November to 6 December and 4 to 17 January are the second darkest 4 weeks of the year and correspond to Index 2. The brightest 4 weeks of the year correspond to Index 13. Figure 1 provides a graphical representation of the Daylight Indices, where the ellipse represents days of the year. Daylight Indices were assigned to each student based on the date of their performance exam. Scores were also analyzed separately as a function of season.

The average national latitudes and longitudes were taken from public online data [18] and used to compare test scores geographically in a different manner than by cluster. Crowding looks at the number of schoolchildren per classroom and is a measure of the schoolchildren/teacher ratio, assuming one teacher per classroom. While crowding does not directly pertain to light, it is a well-known confounder for performance, as is CO_2_, as mentioned above. Window orientation is a binary variable indicating either a view from the classroom onto green space or urban space/other buildings.

The perception of illumination variable is a subjective variable that estimates the overall illumination in the room and was asked of the teachers, schoolchildren, and parents individually. Thus, there are three measures of the same variable to be taken into account when considering any associations with schoolchildren performance test scores.

### 2.3. Performance Tests

Schoolchildren’s performance in the classroom was measured by means of a math and logic test given at the start of the school day. The first section tested basic arithmetic skills, while the second part of the exam focused on logic and memory skills. The second section consisted of 119 logical translation, or ciphering, elements, and children were given 120 s to complete as many as possible. Two slightly different versions of the test (Test 1 (basic arithmetic) and Test 2 (logical)) were administered in different countries. Czech Republic, Italy, and Lithuania Portugal, and Serbia took the second variant of the test whereas other European countries took the first variant of the tests (Figure S1). Both versions of the exam are treated similarly, since the basic content was similar even if individual questions were different. Test scores are based on a total of 200 points for each test (100 points each from Section 1 and Section 2) and were represented by a percentage of correct answers.

### 2.4. Statistical Methods

All light-related variables were initially considered equally as potential indicators of performance. The importance of each individual variable on the average score was determined by either a t-test or ANOVA for categorical variables (e.g., direct sunlight) or by a univariate regression against the average test score. This enabled a better understanding of which indicators might be most relevant and allowed for verification that our data followed known patterns such as a significant dependence on indoor CO_2_ concentrations.

Variables were included in a multivariate analysis if they were significant (*p* < 0.05) in the initial univariate screening process, while irrelevant variables were generally excluded in order to simplify the dataset and focus on variables that might truly impact performance levels. Multiple linear regression analysis was used to determine the overall model and to control for confounding influences.

Finally, window/floor area ratio, type of shading, latitude, percentage of windows facing south, daylight index, direct sunlight, window glazing, and open-able windows were analyzed using multiple linear regression analysis to determine the overall model and to control for confounding influences such as age, gender, race, and maternal education. CO_2_ was also included as it is known to be correlated with lower school performance, and this study also investigated light as a modifier to this effect. The significance level was set at (*p* < 0.05). All statistical analyses were done using Stata 14 (StataCorp. 2015. Stata Statistical Software: Release 14. College Station, TX, USA: StataCorp LP.).

## 3. Results

To exclude any selection effects, no demographic bias was observed in the schoolchildren who participated in the performance exams (Table 2) as compared to that of the wider SINPHONIE study. The ratios of schoolchildren by gender, ethnicity, and education of the mother were similar for test takers as for the total SINPHONIE population as a whole. Here, maternal education level was used as a proxy for socio-economic status. There was a slight shift in the geographical distribution of the schoolchildren, with cluster 2 (Western Europe) representing only 11.46% of the test taking subgroup versus 16.29% of the entire SINPHONIE population. Cluster 3 (Central–Eastern Europe) represented highest number of test takers (38.84%) followed by cluster 4 (36.63%). Northern and Central Eastern Europe represent approximately the same percentage in each sample within ≈2 percentage points difference among test takers and the total population included in the study. Various classroom characteristics such as ceiling height, story number, window area, or floor area were essentially the same for both the test takers and the general SINPHONIE population, and they did not present any indications of bias in the selection of schoolchildren.

### 3.1. Schoolchildren Performance and Test Scores

**Figure 2:**
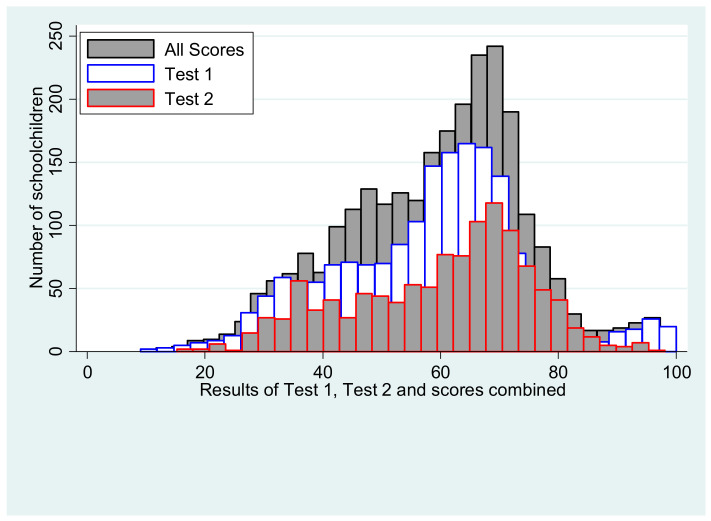
Average performance test scores for both Test 1 and Test 2 and combined.

A total of 1765 children completed Test 1, and 886 children completed Test 2. Each two-section exam was considered as single measure of performance. The average scores are 59.25% (Standard Error, SE = 0.47) and 58.39% (SE = 0.47) for female and male schoolchildren, respectively (Table S2). These average scores and their distributions are shown in the histogram in Figure 2. While the distinction between Test 1 and Test 2 is shown here, the analysis using lighting variables combines all schoolchildren and considers the average of all the test results. Table S2 shows the average scores for each test separately and the combined value according to gender used in the forthcoming analysis. Table S3 indicates the level of performance according to the geographical zone, with Northern Europe having the highest scores, followed by Southern Europe, Western Europe and Central Eastern Europe. Average scores were higher during autumn and winter months, and we found schoolchildren performing best during the month of October (Figure S2).

### 3.2. School Characteristics, Perception of Lighting, and Schoolchildren Performance

Table S4 presents the relationship between test performance exam scores and various classroom characteristics. A positive impact was observed with the perception of illumination of teachers, type of window glazing and shading, as well as with the ability to control the window shading. There is also a slight, but significant, effect of latitude, with higher average scores for more northern countries. No discernable association was observed between the parent’s or children’s perception of illumination, direct sunshine on benches, type of lighting, and open-able windows. Since the associations between the perception of illumination by schoolchildren, parents, and teachers are inconsistent with each other and with other indicators of light such as window area, and because of subjective nature of the question, this variable was left out of the multivariate analysis. It is reported here for the sake of completeness. Figure S2 shows the average test scores by season and months.

### 3.3. Correlation between Average Score and Daylight Parameters

Table S5 shows the correlation between average score and different daylight parameters. We observed no strong correlation among the studied parameters. A statistically significant weak correlation was observed between shading, latitude and average score among schoolchildren.

### 3.4. Relationship between Daylight and Performance

Non-linear regression for the continuous variables did not yield any more detailed results than did linear regression. Several variables show a significant association with average performance test scores with the following exceptions. The window orientation was not significant, suggesting no difference in scores between classrooms facing urban settings and those facing green space. Similarly, the size of the floor area, type of wall covering or writing board, ceiling type or story number of the classroom showed no impact on performance. The percentages of window area facing north, east, or west were not significant; however, the percentage of the window area facing south was significant.

The variables that individually showed a significant influence on the performance test scores are listed in Table 3, and the strength of the associations between each of the significant continuous variables and the mean performance test scores is summarized. The strongest predictor of performance was the window-to-floor area ratio. The strength of this association persisted in a multivariate linear regression analysis, as described in the following section. There is only a slight difference in scores when regressing on window area alone.

Then, variables with significant association in the univariate analyses were analyzed further to understand the overall influence that these indicators have in schoolchildren’s performance.

When all light indicators are included in a single model, the positive influence from the window-to-floor area ratio dominates, followed by the type of shading, the latitude, and finally the percentage of window area facing south. We found that other parameters namely daylight index, direct sunlight, and open-able windows were non-significantly inversely associated with average scores.

A simple linear regression on the average score shows a slight significant negative impact on scores with increasing CO_2_ concentrations; however, this result does not vary for differing values of the window-to-floor area ratio. When regressed separately for different quartiles of window-to-floor area ratio, there was no change to the effect on scores from CO_2_ concentrations (results not shown).

### 3.5. Taking into Account Potential Modifiers

#### 3.5.1. Geographic Cluster

Scores were analyzed within geographic clusters to understand any difference between regions. The mean test scores for each cluster are provided in Table S3, which shows that the schoolchildren in cluster 1 outperform those in clusters 2, 3, and 4 by approximately 16 percentage points, whereas similar scores were observed across the other three clusters.

When the multivariate model was run for each cluster individually, results vary depending on the cluster. Only cluster 3, with the highest number of test takers, and cluster 1 show models similar to the overall multivariate model found for the study as a whole. These are also the only clusters where the window-to-floor area is a significant positive indicator of school performance.

Other variables, such as the percentage of windows facing south, direct sunlight, the average national latitude, or the ability to open windows change their direction of impact depending on the cluster. Some variables have an impact in some clusters but not in others, such as the type of glazing (results not shown). In general, there is no consistent aspect of the models between clusters, and more information would be necessary to try to understand why the various indicators impact scores positively in one case but negatively in another.

#### 3.5.2. Gender

Models were also run separately for male and female schoolchildren. When the same multivariate regression is run for each gender separately, we see that window-to-floor area is not an important factor for females; however, the rest of the variables predict performance in much the same way for all schoolchildren. The coefficients for each indicator other than window-to-floor area are roughly the same for both males and females (results not shown).

#### 3.5.3. Season

Test scores vary by season, with schoolchildren taking performance tests in the summer performing best (average score = 60.66%) and schoolchildren taking tests in the spring performing worse (54.76%). Schoolchildren performing the tests in winter have an average score of 58.77. Figure S2 shows these values graphically, as well as the average score by month. However, not all clusters took exams during all seasons, and only cluster 3 took exams in the spring, which may impact results.

Different results are obtained when the multivariate regression is performed by season. However, the regression failed when only scores from spring were included (Daylight Index was left out when regressing by season). In both autumn and winter, the window-to-floor area ratio still dominates; however, ceiling height, open-able windows, and the percent of windows facing south seem to have a positive impact in one season and a negative impact in the other. The only other indicator consistent between the two seasons is the average national latitude (results not shown).

## 4. Discussion

The main objective of our study was to investigate whether school performance is affected by lighting conditions inside classrooms in Europe. Our data is drawn from a population-based sample of primary schoolchildren in 53 schools from 12 European countries and shows a significant relationship between light-related variables and school performance. The window-to-floor area ratio is a highly significant indicator of school performance, indicating that a larger window area for a given room size has a positive impact on grades and achievement. This variable remains the strongest predictor when adjusting for confounders such as gender. However, it is not consistently important over the geographic region, and it does not seem to be as important for females as it is for males. In terms of gender, one can only speculate why window size might be more important for male students than for female students. This could be due to hormonal differences influencing cognition or in aptitudes between boys and girls. However, in terms of geography, we see that the window-to-floor area impacted schoolchildren in cluster 4 more than the others. These are the southernmost European countries, were there is more consistent sunlight, and schools may be more reliant on natural light vs. artificial light. In general, there is no consistent aspect of the models between clusters, and more information would be necessary to try to understand why the various indicators impact scores positively in one case but negatively in another.

The type of window shading also appeared to be significant, and it does not seem to be correlated with direct sunlight. Classrooms with internal shading and south side shading had significantly higher average scores than did those with external or no shading, suggesting that it is advantageous to control the amount of sunlight entering the classroom, especially if glare is indeed a problem for some classrooms, as implied by the fact that we found a negative association with direct sunlight on exam performance. A similar negative association between direct sunlight was seen by [6], who found that schoolchildren in classrooms with diffuse natural light performed better than those with direct light. When all light indicators are included in a single model considering also the latitude other parameters like the daylight index and open-able windows were non-significantly inversely associated with average scores. This could be due to statistical problems due to the fact that all the light indicators are related to each other. However, this lack of significance needs to be acknowledged and investigated in further studies.

Overall, schoolchildren performed better in the summer and winter. Lower scores in the spring may be a result of general burn-out over the course of the school year, although higher scores in the summer may contradict that theory. It is general knowledge that schoolchildren in Northern Europe perform better than Southern and Eastern Europe and indeed better than many countries around the world. The PISA ranking for 2018 [19] supports our result that exam scores are higher in Northern European countries.

Ceiling height was significant when regressed directly against the mean performance test scores with a univariate model, but it did not remain significant when other light indicators were included. We observed schoolchildren performing better in tests with triple glazed windows but did not see a significant difference in average scores between single- or double-glazed windows and double-glazed windows with filling. Triple glazed windows may also be a sign of affluent neighborhoods or colder, northern latitudes, rather than an effect due to the glazing itself, which could confound the results. There was no observable impact on performance from the orientation of the windows, suggesting no difference between a view toward green space or toward urban space. However, it should be noted that window height was not included in the data, and it is impossible to know if the schoolchildren were able to see out the windows from their seats.

Perception of illumination was excluded from the multivariate analysis on the basis of subjectivity, as well as inconsistencies between the schoolchildren, parents, and teachers. However, it could be argued that the use of teachers’ perception of illumination, which was a significant predictor for performance, is justified, as their judgment should be based on a full day’s light from an adult with an understanding of differences between classrooms and various lighting conditions.

### 4.1. Potential Mechanisms

Several potential mechanisms have been suggested concerning the impact of lighting conditions inside schools and offices and its effect on performance among building occupants [14,20]. Included amongst them are increased visibility, enhanced mood and improved health and shading conditions, reduction from flickering effects from electric lighting, and better mental alertness due to circadian responses to daylight.

### 4.2. Strengths and Weaknesses

The major strength of the present study is that the data are drawn from the general population of primary schoolchildren, spanning more than 50 schools and 155 different classrooms in 12 countries across all regions of Europe. Compared to studies focused only on a handful of classrooms or schools, with a sample size of 2670 students, our study is able to observe daylighting effects that are more general and not specific to one location. We were also able to control for several confounding factors such as age, gender, maternal education, as well as for CO_2_ level, which could significantly affect the associations as currently observed, the effects of CO_2_ on mental performance being well documented.

A major limitation of our study is the fact that light was not originally considered as a stressor in the implementation of the SINPHONIE design protocol. As a consequence, no direct assessment of light was performed, and exposure misclassification cannot be excluded because light was not determined objectively. While this may limit the comparison between previous studies, there is still considerable data within the SINPHONIE study related to classroom lighting conditions to warrant an investigation. As a consequence of the fact that light was not one of the research questions of the study, bias toward light assessments is not expected, and classrooms were not selected based on lighting conditions. Similarly, the repertoire of cognitive tasks appears way too narrow for a reliable assessment of cognition. More specific tasks for attention and performance such as attention network test or more complex cognitive functions such as go/no go task would have provided with a better interpretation of the results, warranting cautious interpretation of the results. A further methodological limitation of the study is its cross-sectional design, which does not allow for inferring causality.

Other limitations result from the differences in school systems for different countries. As a result of this heterogeneity, comparisons have to be made cautiously. Additionally, our analysis could not take all potential confounders of the relationship into account, such as air pollution, noise levels, and the use of screens can impact learning and impinge on the school performance. Socio-economic status, another confounder, was considered, but through the proxy of maternal education level. Another major confounder that we were unable to control for is the sleepiness levels; information on sleep-wake diaries could have provided a better picture of the effect of cognitive performance. However, to avoid sleepiness, most performance tests were performed in the morning.

Exposure to indoor air pollution and other demographic factors have also been associated with the decrease in learning and performance in schoolchildren [21]. In addition, while daylight has significant benefits, access to open windows can also have unintended side effects such as leaking ambient air pollution inside the rooms [22].

## 5. Conclusions

For the first time in Europe, we related several parameters associated with daylighting to mathematics and logical test results in a large sample of schoolchildren. Overall, our findings suggest that classroom characteristics associated with daylighting do significantly impact the performance of the schoolchildren and may account for more than 20% of the variation between performance test scores. The window-to-floor area ratio in classroom appears to have the largest effect, indicating that larger window areas are advantageous. Having control over the shading of the windows is also important, most likely because it is indirectly related to sunlight. Scores are higher for tests taken during summer and winter months. Further studies are needed to confirm our findings.

## Figures and Tables

**Table 1:** Collected data relevant to lighting.

Variable Type	Variable	Unit or Category
**Continuous**	Ceiling Height	m
	Floor Area	m^2^
	Window Area	m^2^
	CO_2_ Concentration	ppm
	Windows Facing North	% of total area
	Windows Facing South	% of total area
	Windows Facing East	% of total area
	Windows Facing West	% of total area
	Window-to-Floor Area Ratio	-
	Average National Latitude	degrees
	Average National Longitude	degrees
	Crowding	Number of schoolchildren
**Categorical**	Perception of Illumination	scale of 0 to 6
	Standard Orientation	Facing Street | Facing Garden
	Wall Covering	Wall Paper | Water-Resistant Paint Whitewash | Water-Soluble Paint
	Standard Board	Blackboard | Whiteboard
	Direct Sunshine	Yes | No
	Number of stories	0,1,2,3
	Type of Lighting	Artificial | Mixture | Natural
	Type of Window Glazing	Single | Double | Triple | Double with Filling
	Type of Window Shading	Internal | External | None
	Control of Window Shading	Individual | No Control
	Suspended Ceiling	Yes | No
	Open-able Windows	All | Some | No
	Daylight Index	-
	Season	-

**Table 2:** Demographic information on performance test takers within the SINPHONIE Study (N = 2760).

	All Test Takers N (%)	Entire Study N (%)
Number of Schoolchildren	2670	5175
Age Range	8–13	3–14
Gender		
Male	1315 (49.25%)	2588 (50.01%)
Female	1336 (50.04%)	2557 (49.41%)
Missing	19 (0.71%)	30 (0.58)
Total	2670	5175
Ethnicity		
Caucasian	1752 (65.54%)	3608 (69.72%)
Asian	44 (1.65%)	84 (1.62%)
Middle Eastern	29 (1.09%)	75 (1.45%)
Black	36 (1.35%)	76 (1.47%)
Other	341 (12.77%)	529 (10.22%)
Missing	470 (17.60%)	803 (15.52%)
Total	2670	5175
Maternal Education		
College/University	1032 (38.65%)	2158 (41.70%)
Secondary School	959 (35.92%)	1768 (34.16%)
Primary School	270 (10.11%)	406 (7.85%)
Less than Primary School	47 (1.76%)	102 (1.97%)
Vocational School	282 (10.56%)	602 (11.63%)
Missing	80 (3.00%)	139 (2.69)
Total	2670	5175
Paternal Education		
College/University	864 (32.36%)	1800 (34.78%)
Secondary School	865 (32.40%)	1598 (30.88%)
Primary School	284 (10.64%)	440 (8.50%)
Less than Primary School	36 (1.35%)	63 (1.22%)
Vocational School	443 (16.59%)	913 (17.64%)
Missing	178 (6.67%)	361 (6.98%)
Total	2670	5175
Number of Schools	53	115
Number of Classrooms	155	319
Number of Countries	12	23
Clusters		
Northern Europe	349 (13.07%)	618 (11.94%)
Western Europe	306 (11.46%)	843 (16.29%)
Central–Eastern Europe	1037 (38.84%)	1865 (36.04%)
Southern Europe	978 (36.63)	1849 (35.73%)
Missing	-	-
Total	2670	5175

The level of schooling refers to the mother of each schoolchildren, which is used as a proxy for socio-economic status.

**Table 3:** Strength and significance of the association between the continuous lighting indicators and the performance test mean score. The coefficient represents the strength of association.

Variable	Coefficient	SE	t	*p*	CI (95%)	
Window/Floor Area Ratio	23.51	3.62	6.5	<0.01	16.41	30.60
Type of Shading	6.64	0.52	12.88	<0.01	5.63	7.65
Latitude	1.18	0.08	15.11	<0.01	1.03	1.34
Percentage of Windows facing South	0.04	0.01	3.51	<0.01	0.02	0.06
Daylight Index	−0.25	0.16	−1.57	0.12	−0.57	0.06
Direct Sunlight	−0.002	0.87	0	1.00	−1.70	1.70
Glazing	3.41	0.50	6.84	<0.01	2.44	4.39
Open-able Windows	0.57	0.38	1.49	0.14	−0.18	1.32

Adjusted on age, gender, race, and maternal education.

## Supplementary Materials

The following are available online at https://www.mdpi.com/1660-4601/18/1/258/s1, Figure S1: Example of the Performance Test given within SINPHONIE study, Figure S2: Average Performance Test Scores by Season (left) and by Month (right), Table S1: Participating countries in the SINPHONIE project with performance exam scores., Table S2: Performance test scores by test and combined, Table S3 Mean test scores by cluster, Table S4: Relationship between test performance scores and various classroom characteristics. Table S5: Correlation between average score and daylight parameters.
