# Supplementary material for: Daylight and School Performance in European Schoolchildren
Source: Int J Environ Res Public Health. 2020 Dec 31;18(1):258. doi: 10.3390/ijerph18010258 (PMC7795157; doi:10.3390/ijerph18010258)

<u>Supplementary Material</u>

Daylight and school performance in European schoolchildren

Ramen Munir Baloch, MPH [1], Cara Nichole Maesano, PhD [1], Jens Christoffersen, PhD [2], Corinne Mandin, PhD [3], Eva Csobod, PhD [4], Eduardo de Oliveira Fernandes, PhD [5], Isabella Annesi-Maesano, PhD, [1]

On behalf of the SINPHONIE Consortium

Table of Contents

Table S1: Participating countries in the SINPHONIE project with performance exam scores.

Table S2: Performance test scores by test and combined.

Table S3: Mean test scores by cluster.

Table S4: Relationship between test performance scores and various classroom characteristics.

Table S5: Correlation between average score and daylight parameters.

Figure S1: Example of the Performance Test given within SINPHONIE study.

Figure S2: Average Performance Test Scores by Season (left) and by Month (right).

Table S1: Participating countries in the SINPHONIE project with performance exam scores.

	Test 1	Test 2
**Countries**	Cluster 4: Albania (n = 438)<br>Cluster 2: Belgium (n = 104)<br>Cluster 3: Bosnia & Herzegovina (n = 133)<br>Cluster 1: Estonia (n = 175)<br>Cluster 2: France (n = 202)<br>Cluster 4: Greece (n = 90)<br>Cluster 3: Hungary (n = 385)	Cluster 3: Czech Republic (n = 272)<br>Cluster 4: Italy (n = 236)<br>Cluster 1: Lithuania (n = 174)<br>Cluster 4: Portugal (n = 214)<br>Cluster 3 : Serbia (n = 247)

Table S2: Performance test scores by test and combined.

	**Test 1**		**Test 2**		**Combined**	
	Female<br>n (%)	Male<br>n (%)	Female<br>n (%)	Male<br>n (%)	Female<br>n (%)	Male<br>n (%)
Number of schoolchildren	894 (50.65%)	871 (49.35%)	442 (49.89%)	444 (55.11%)	1336 (50.40%)	1315 (49.60%)
Mean test score*	57.10	56.56	63.60	61.98	59.25	58.39
Standard error	0.58	0.59	0.72	0.73	0.47	0.47

*Mean test score uses the percentage of correct answers

Table S3: Mean test scores by cluster.

Cluster	Geographical zone	Mean Performance Test Score	Standard Deviation	Frequency
**1**	Northern Europe	72.20	15.78	349
**2**	Western Europe	56.40	13.94	306
**3**	Central-Eastern Europe	56.37	18.56	1037
**4**	Southern Europe	56.98	13.87	978

Table S4: Relationship between test performance scores and various classroom characteristics.

Variable	Association with Exam Scores
Perception of Illumination - Teachers	Positive impact on score with increased perception
Perception of Illumination - Schoolchildren	No discernible association
Perception of Illumination - Parents	No discernible association
Direct Sunshine	No discernible association
Type of Lighting	No discernible association
Type of Window Glazing	Positive impact on score with triple glazing.
Type of Window Shading	Positive impact on score with South side shading.
Control of Window Shading	Positive impact on score with control of shading
Open-able Windows	No discernible association found

Table S5: Correlation between average score and daylight parameters.

Average score	Window/ floor area ratio	Type of shading	Latitude	Percentage of windows facing South	Daylight index	Direct sunlight	Glazing	Ceiling height	Open-able windows	Open-able windows
Average score	1									
Window/ floor area ratio	0.1559	1								
Type of shading	0.2454	0.1196	1							
Latitude	0.3084	0.1651	0.2154	1						
Percentage of windows facing South	0.0888	0.0721	0.1543	0.3042	1					
Daylight index	-0.0329	0.0752	-0.011	-0.0722	-0.037	1				
Direct sunlight	-0.0075	0.0446	-0.059	0.2001	0.0674	-0.056	1			
Glazing	0.0917	-0.0242	0.0755	-0.2261	-0.058	-0.06	-0.081	1		
Ceiling height	-0.0513	0.0879	-0.174	-0.159	-0.089	0.3469	0.0419	0.0487	1	
Open-able windows	0.0221	0.0479	0.1606	0.0201	-0.086	-0.186	-0.09	0.1858	-0.2084	1

Figure S1: Example of the Performance Test given within SINPHONIE study.

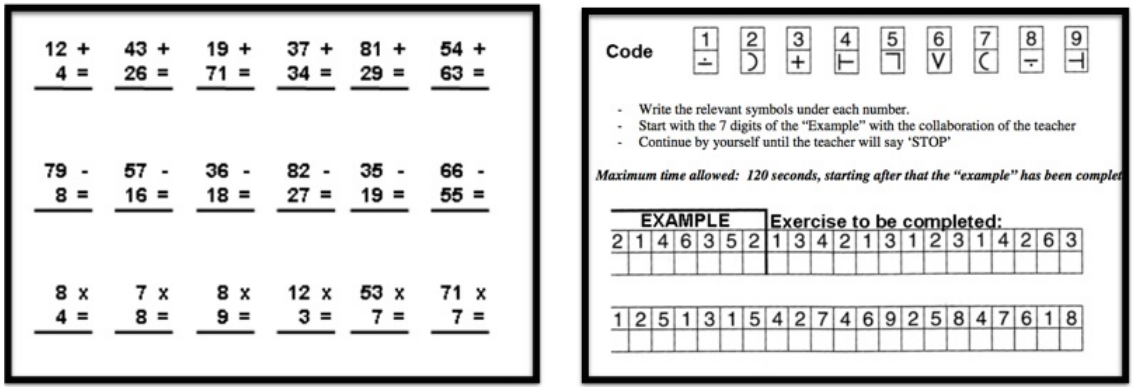

Section 1 (left) included basic arithmetic, while section 2 (right) tested ciphering skills and memory.

Figure S2: Average Performance Test Scores by Season (left) and by Month (right).

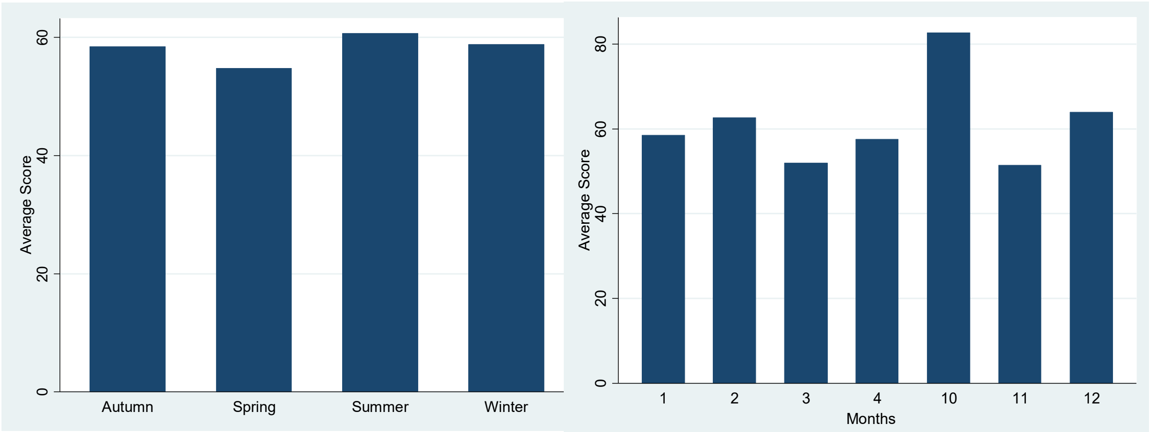

Supplement: Supplementary file 1 [file ijerph-18-00258-s001.pdf]
